# Predictive Value of Serum miR-10b, miR-29c, and miR-205 as Promising Biomarkers in Esophageal Squamous Cell Carcinoma Screening

**DOI:** 10.1097/MD.0000000000001558

**Published:** 2015-11-06

**Authors:** Hang Xu, Yuanfei Yao, Fanyu Meng, Xu Qian, Xiaofeng Jiang, Xiaoxi Li, Zhuo Gao, Lu Gao

**Affiliations:** From the Department of Pharmacy, Nanjing Drum Tower Hospital, the Affiliated Hospital of Nanjing University Medical School, Nanjing, China (HX); School of Life Science and Technology, Harbin Institute of Technology, Harbin, China (YY); School of Public Health, Harbin Medical University, Harbin, China (FM); Department of Neuro-Oncology, U.T. M.D. Anderson Cancer Center, Houston, TX, USA (XQ); Department of Clinical Laboratory, the Fourth Affiliated Hospital of Harbin Medical University, Harbin, China (XJ, ZG); The Center of Metabolic Disease Research, Nanjing Medical University, Nanjing, China (XL, ZG); Department of Surgery, University of Maryland School of Medicine, Baltimore, MD, USA (ZG); College of Life Sciences, Northeast Agricultural University, Harbin, China (LG); and Department of Pathology, University of Maryland School of Medicine, Baltimore, MD, USA (LG).

## Abstract

Esophageal squamous cell carcinoma (ESCC) is a leading cause of cancer-related deaths worldwide. The high mortality of ESCC is mainly due to late diagnosis. Current detection methods have their own weakness, including high costs and invasive procedures. MicroRNA assays are shown to have great potential to be accurate and noninvasive methods for ESCC screening. In this study, we selected 3 microRNAs, miR-10b, miR-29c, and miR-205, to assess their diagnostic value in ESCC screening. Fifty ESCC patients and 50 healthy controls are recruited in our study. Blood samples are collected from the total 100 participants. MicroRNAs were extracted from serum and quantified by qRT-PCR, which their relative expressions were normalized by internal control, U6 snRNA. Statistical analyses were conducted to compare microRNAs level as well as other clinical characteristics between 2 groups. The levels of serum miR-29c and miR-205 were significantly downregulated in ESCC patients compared with healthy volunteers. In contrast, ESCC patients appeared to have a higher level of miR-10b than healthy controls. ROC curve analyses revealed that the AUC value for miR-10b, miR-29c, and miR-205 were 0.85 (95% CI: 0.79–0.93; sensitivity = 76%; specificity = 84%), 0.72 (95% CI: 0.62–0.82; sensitivity = 68%; specificity = 68%), and 0.72 (95% CI: 0.62–0.83; sensitivity = 70%; specificity = 64%), respectively, suggesting that miR-10b, miR-29c, and miR-205 have great potential to be noninvasive screening tools for ESCC detection.

## INTRODUCTION

Oesophageal cancer is the 8th most frequently occurring cancer and the 6th major cause of cancer-related mortality worldwide, with approximately 456,000 new cases and 400,000 cancer-related deaths in 2012.^1^ An estimated 80% of the cases occur in underdeveloped countries, particularly in China. China, with the high morbidity rate and large population, accounts for more than half of oesophageal cancer-related deaths in the world.^2^ According to the histological features of malignant cells, the oesophageal cancer can be divided into 2 major types, oesophageal adenocarcinoma (EAC) and oesophageal cell squamous carcinoma (ESCC). ESCC is one of the most aggressive carcinoma of gastrointestinal tract with poor prognosis, which dominates almost 90% of oesophageal cancer cases worldwide.^3^ Despite the progress in clinical treatment, the overall 5-year survival rate of ESCC patients still remains low (around 10%) largely due to delayed diagnosis and high recurrence rate.^4^ Therefore, early detection of primary tumors provides opportunities to implement effective treatments and timely interventions to optimize patient outcomes.

The medical imaging techniques, including endoscopy and X-ray, are regarded as the powerful tools to detect ESCC, which have been already widely applied in the clinical diagnosis. However, there are still some limitations on these powerful tools. For instance, an endoscopy is required to insert into mouths and move toward stomach to visualize the signs of abnormal cells or tumors, which may cause discomfort or even pain in patients during the procedure. The X-ray system might pose a potential radiation risk to patients. Currently, the molecular biomarkers as the promising noninvasive diagnostic approaches have been widely investigated in recent studies. Several molecular targets, such as COX-2, EGFR, VEGF, p16 and FAS, etc., are clarified to play important role in carcinogenesis and progression of cancer. However, there are only a few molecules that have been clinically validated as diagnostic biomarker for ESCC.^5^ Conventional tumor markers, such as carcinoembryonic antigen (CEA), E-cadherin, CA-125, and alpha-fetoprotein, have been successfully employed as a convenient diagnostic assays to detect certain cancers.^6^ Nonetheless, these tumor markers cannot provide sufficient sensitivity and specificity in early-stage ESCC detection.^7,8^ Thus, there is an urgent need to identify the novel accurate biomarkers with less invasiveness for the early-stage ESCC diagnosis.

MicroRNAs are a large family of small noncoding RNAs that regulate posttranscriptionally the gene expression by binding to the 3′-untranslated region (UTR) of messenger RNA (mRNA), leading to translational repression. It is estimated that up to 30% of the protein-coding genes are regulated by a single RNA.^9^ As a result, microRNAs are involved in diverse biological processes including cell proliferation, differentiation, and apoptosis. A number of studies have indicated that the abnormal microRNAs expression is associated with initiation and development of cancer.^10^ In addition, miRNAs are shown to be detectable in cell-free body fluids, such as serum, plasma, blood, urine, and feces. It is demonstrated that miRNAs exhibit high stability under extreme conditions such as endogenous ribonuclease activity, high/low pH, boiling as well as multiple freeze thaw cycles.^11^ In view of the advantages mentioned above, miRNAs have great potential to be useful biomarkers in cancer detection.

In previous studies, miR-10b is reported to be significantly elevated in both human ESCC tissues and metastatic breast cancer cells. MiR-10b inhibits Kruppel-like factor 4 (KLF4) in ESCC, resulting in cancer migration and invasion.^12^ Similarly, miR-10b can negatively regulate the HOXD10 in breast cancer, leading to overexpression of prometastatic gene RHOC.^13^ Moreover, miR-29c, which acts as a tumor suppressor by targeting the oncogene SIRT1, is downregulated in hepatocellular carcinoma.^14^ The decreased expression of miR-29c is detected in ESCC tumor tissues as well.^15^ Besides, low level of miR-205 may induce the invasion and migration of ESCC cells.^16^ The tumor-suppressive activity of miR-205 is also observed in lung and breast cancer.^17,18^

In present study, we selected 3 candidate microRNAs (miR-10b, miR-29c, and miR-205) to assess their diagnostic values in ESCC screening by comparing their expression level in serum between ESCC patients and healthy volunteers.

## MATERIALS AND METHODS

### Patients and Samples

The research was conducted in strict accordance with the protocol approved by the Ethics Committee of the Fourth Affiliated Hospital of Harbin Medical University, and a written informed consent must be provided by each participant. From 2010 to 2013, a total of 100 participants from the Fourth Affiliated Hospital of Harbin Medical University, including 50 ESCC patients and 50 healthy controls, were enrolled in our study. Each enrolled patients has to meet the following criteria: diagnosis of ESCC was clinically confirmed by histopathology or biopsy; patients have no previous history of malignancy; patients have not received chemotherapy, radiotherapy, or operation. The stage of tumors was determined according to the tumor–node–metastasis (TNM) staging system. The characteristics of patients in regards to age, gender, and other clinical characteristics were described in Table 1.

**TABLE 1:**
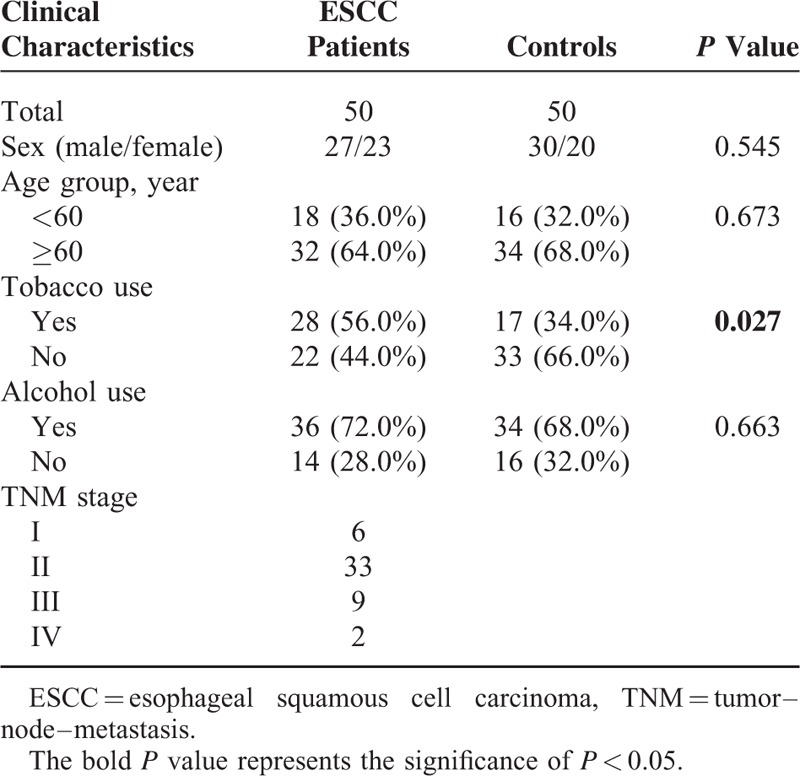
Comparison of ESCC Patients and Controls by SelectiveCharacteristics

### RNA Extraction

Blood samples were collected from each patient and were initially isolated into serum and cellular sediment by centrifugation at 3000 rpm for 10 minutes. The supernatant was collected and followed by further centrifugation at 12,000 rpm for 5 minutes to obtain the serum. All serum samples were stored at −80 °C before further analysis. The extraction of total RNA from 400 μL blood serum was performed using mirVana PARIS Kit (Ambion, Austin, TX) according to the manufacturer's instructions. Total RNA was eluted by 50 μL RNase-free water (Ambion, Austin, TX). The concentration and purity of the RNA solution was measured by detecting its absorbance at 260/280 and 260/230 nm with NanoDrop 1000A spectrophotometer (NanoDrop Technologies, Wilmington, DE). All the purified RNA samples were stored at −80 °C for further processing.

### Quantitative Reverse-Transcriptase Polymerase Chain Reaction (qRT-PCR)

The qRT-PCR was conducted to quantify the amount of miRNA by using human TaqMan MicroRNA Assay Kits (Applied Biosystems, Foster City, CA). The reverse transcription reaction was performed with TaqMan MicroRNA Reverse Transcription Kit (Applied Biosystems) which has a high specificity. For cDNA synthesis, the reaction mixture was prepared, which contained 5 μL of RNA extract, 3 μL of reverse transcription primers, 0.15 μL of 100 mM dNTPs, 1.5 μL of 10× reverse transcription buffer, 0.19 μL of 20 U/μL RNase inhibitor, 1 μL of 50 U/μL multiscribe reverse transcriptase, and 4.16 μL nuclease-free water. The reaction solutions were incubated at 16 °C for 30 minutes, subsequently at 42 °C for 30 minutes, and then at 85 °C for 30 minutes, and finally held at 4 °C. To prepare the qRT-PCR reaction solutions, 1.33 μL cDNA after reverse transcription reaction is mixed with 10 μL of TaqMan Universal PCR Master Mix II (2×) with no UNG reagent (Applied Biosystems), 1 μL of specific primers and 7.67 μL of RNase-free water in a total volume of 10 μL. The qRT-PCR was carried out on a Bio-Rad IQ5s system (Bio-Rad Laboratories, Inc.). Shortly after an initial denaturation at 95 °C for 10 minutes, the amplification was repeated for 45 cycles at 95 °C for 15 seconds and at 60 °C for 60 seconds. The cycle threshold (Ct) values were calculated by Bio-Rad IQ5 2.1 Standard Edition Optical System Software 2.1.94.0617. U6 snRNA was used as the reference gene to determine the relative expression level of miRNAs. The change in miRNA expression is defined as 2^−ΔΔCt^, which ΔCt represented the difference of Ct values between miRNAs and U6 snRNA, ΔΔCt was the difference of ΔCt values between healthy and ESCC samples.

### Statistical Analysis

The difference in miRNAs levels between the cancer and healthy groups was analyzed by student's paired *t*-test. A *P* value less than 0.05 was considered as statistically significant. Receiver operating characteristic (ROC) curves were constructed based on the miRNA levels between groups. Area under the ROC curve (AUC) was generated to assess the diagnostic values of the candidate microRNAs. The cutoff values for microRNA levels were determined by Youden index. All the statistical tests were conducted by STATA version 12.0 software (Stata Corp, College Station, TX), and the graphs were obtained from GraphPad Prism 5.0 (GraphPad Software Inc., CA)

## RESULTS

### Clinical Characteristics of Study Population

Serum samples were acquired from 100 subjects (50 ESCC patients, 50 healthy controls). As presented in Table 1, no significant difference in age, gender as well as consumption of alcohol was observed between cancer and healthy group (*P* > 0.05). However, there was apparent difference in tobacco use between ESCC patients and healthy controls (*P* = 0.027). Among 50 ESCC patients, 6 were diagnosed as Stage I ESCC, 33 as stage II ESCC, 9 as stage III ESCC, and 2 as stage IV ESCC.

### MicroRNAs Level and ESCC Susceptibility

**FIGURE 1:**
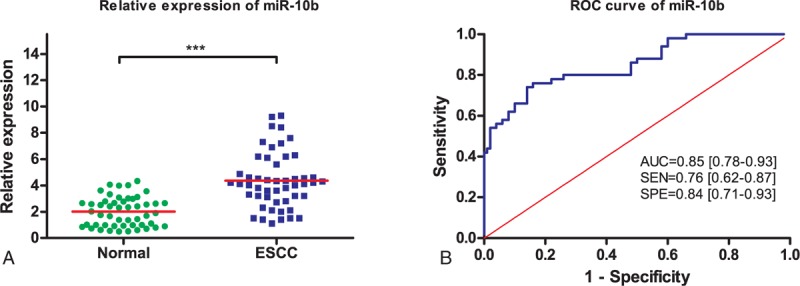
Schematic diagram depicting microRNAs dysregulation in esophageal squamous cell carcinoma (ESCC). MicroRNAs dysregulation may result in ESCC formation and progression through affecting the expression of target genes and then the progress of cell cycle, apoptosis, and tumor invasion and metastasis of ESCC patients.

**FIGURE 2:**
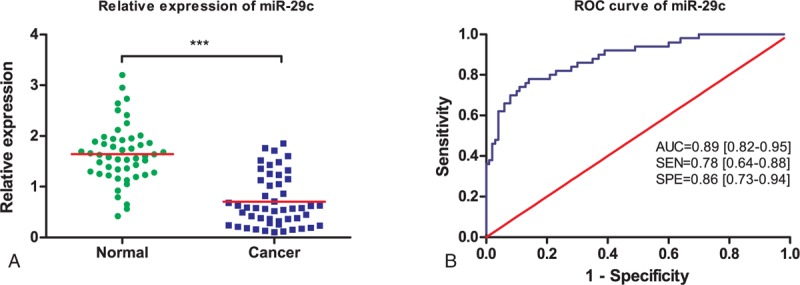
Diagnostic performance of serum miR-10b in differentiating ESCC patients from healthy controls. (A) Relative expression levels of serum miR-10b in ESCC patients and healthy controls. (B) ROC curve analysis of serum miR-10b in differentiating ESCC patients from healthy controls. ESCC = esophageal squamous cell carcinoma, ROC = receiver operating characteristic.

**FIGURE 3:**
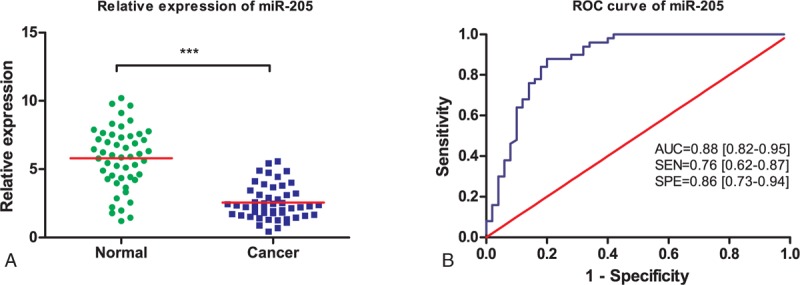
Diagnostic performance of miR-29c in differentiating ESCC patients and healthy controls from ESCC patients. (A) Relative expression levels of miR-29c in ESCC patients and healthy controls. (B) ROC curve analysis of miR-29c in differentiating ESCC patients from healthy controls. ESCC = esophageal squamous cell carcinoma, ROC = receiver operating characteristic.

Three microRNAs (miR-10b, miR-29c, and miR-205) were examined in serum samples from 50 ESCC patients and 50 healthy volunteers. Serum levels of miR-10b, miR-29c, and miR-205 were plotted in the form of scatter dots in Figures 1A, 2A, and 3A. As shown, expression of miR-29c and miR-205 were significantly downregulated in ESCC patients (*P* < 0.001), compared with the healthy volunteers. However, overexpression of miR-10b is detected in ESCC patients (*P* < 0.001). Otherwise, the expressions of 3 microRNAs in tobacco use between ESCC patients and healthy controls have no significant difference (*P* > 0.05). Hence, these 3 miRNAs were able to discriminate ESCC patients from healthy controls based on their aberrant expression patterns.

### Diagnostic Performance of MicroRNAs in ESCC Detection

We further evaluated the diagnostic value of 3 selected miRNAs by ROC curves and AUC values. ROC curves were illustrated in Figures 1B, 2B, and 3B. It was shown that the AUC value for miR-10b, miR-29c, and miR-205 were 0.85 (95% CI: 0.78–0.93; sensitivity = 76%; specificity = 84%; Fig. 1B), 0.89 (95% CI: 0.82–0.95; sensitivity = 78%; specificity = 86%; Fig. 2B), and 0.88 (95% CI: 0.82–0.95; sensitivity = 76%; specificity = 86%; Fig. 3B), respectively.

## DISCUSSION

Several protein biomarkers were identified for ESCC, such as dikkopf-1,^19^ fascin, and galectin-7.^20,21^ However, due to their limited accuracy of diagnosis, they are not appropriate for the use of clinical diagnosis for ESCC, compared with miRNAs. In this study, we demonstrated dysregulation of miR-10b, miR-29c, and miR-205 between the ESCC patients and healthy volunteers. MiR-29c and miR-205 were notably under-expressed in patients, whereas the expression of miR-10b was upregulated in patients. Collectively, our study provides evidences that serum level of miR-10b, miR-29c, and miR-205 have great clinical value as promising biomarkers in ESCC preliminary screening.

Recent studies have been performed to explore the mechanism how miRNAs are implicated in cancer initiation and development, and to assess the potential of miRNAs as therapeutic or diagnostic targets in cancer detection. Ren et al^22^ have demonstrated that miR-183 might promoted ESCC cell proliferation and invasion by binding to the 3′-untranslated region, which has a fundamental function in the development and progression of ESCC. In this study, miR-10b, miR-29c, and miR-205 were selected as potential tumor markers in our study. It is reported that increased expression of miR-10b is associated with breast cancer metastasis. Patients with higher miR-10b level are more likely to progress, develop breast cancer metastasis.^23–25^ Moreover, miR-10b, expressed differentially in pancreatic cancer patients, has shown to promote pancreatic cancer cell proliferation and tumor growth.^26,27^ High level of miR-10b may contribute to neurofibromatosis type 1 (NF1) tumorigenesis and progression through targeting neurofibromin and RAS signaling.^28^ Few researches have previously reported the relation between miR-10b expression pattern and ESCC. These studies demonstrated the oncogenic role of miR-10b in a variety of cancer, which was also consistent with our findings. Emerging evidences show that miR-29c serves as tumor suppressor in carcinogenesis. A decrease in miR-29c expression has been identified in different tumors, including breast, liver, stomach, nasopharynx, skin, and colon cancers.^14,29–33^ In our results, we further validate the tumor-suppressive function of miR-29c in ESCC. MiR-205 inhibits VEGF-A and further suppresses human glioblastoma cell growth.^34^ As for colorectal cancer, miR-205 can be used as biomarker to differentiate lymph node metastatic colorectal cancers from nonmetastatic colorectal cancers.^35^ MiR-205 is significantly decreased in gastric cancer patients compared with healthy controls.^36^ Our study revealed a low level of miR-205 in ESCC patients as well. Taken together, these studies suggest the possibility that miR-205 could serve as a potential biomarker for cancer detection.

Several microRNAs have been shown to be involved in ESCC development. Low expression of miR-100 suggested a potential function on the aggressive tumor progression and unfavorable prognosis of ESCC patients.^37^ By contrast, upregulated miR-183 level compared with adjacent normal control tissues directly inhibits programmed cell death 4 (PDCD4), which could lead to ESCC cell proliferation, migration, and invasion.^38^ Additionally, SOX6, a reportedly tumor suppressor, is repressed by miR-208. The overexpression of miR-208 might result in ESCC cell proliferation.^39^

The validity of RT-PCR results depends on the accurate normalization of microRNAs levels. Therefore, the selection of internal control may have influence on the results. In this study, we selected U6 snRNA as internal control. U6 snRNA is known to have a constant expression in patients and controls. Certain limitations in our study should be addressed as follows. The population of enrolled patients and controls was relatively small. Further study on a larger sample is needed to confirm our results. In terms of ethnicity, the research was conducted in the Asian population. Hence, it remains unknown whether 3 microRNAs have the same diagnostic performance in Caucasian or African group. Subgroup analysis on ethnicity is recommended in further study.

In conclusion, despite the limitations, our findings revealed differential expression of serum miR-10b, miR-29c, and miR-205 in ESCC patients and indicated the important role of their dysregulation in ESCC initiation and progression. Thus, miR-10b, miR-29c, and miR-205 have great potential to be noninvasive screening tools for ESCC detection. A large-scale investigation is required to further validate their diagnostic accuracy before future application in clinical settings.
