# Predictive Value of Serum miR-10b, miR-29c, and miR-205 as Promising Biomarkers in Esophageal Squamous Cell Carcinoma Screening: Erratum

**DOI:** 10.1097/01.md.0000476363.62356.5b

**Published:** 2015-12-31

In the article “Predictive Value of Serum miR-10b, miR-29c, and miR-205 as Promising Biomarkers in Esophageal Squamous Cell Carcinoma Screening”,^1^ which appeared in Volume 94, Issue 44 of *Medicine*, Dr. Xiaoxi Li's name was omitted from the byline. In addition, the article did not correctly report the funding associated with the study. The article has since been corrected online.
